# Laser-Assisted Endoscopic Cricotracheal Stenosis Resection (CTSR) in Paediatric Congenital Cartilaginous Subglottic Stenosis

**DOI:** 10.1155/2020/9528249

**Published:** 2020-06-24

**Authors:** Jaber Alshammari, Abdullah Arafat, Mohammed Halawani

**Affiliations:** Division of Otolaryngology Head & Neck Surgery, King Abdullah Specialized Children Hospital (KASCH), King Abdulaziz Medical City (KAMC), National Guard Health Affairs (NGHA), Riyadh, Saudi Arabia

## Abstract

Subglottic stenosis (SGS) in children can be a congenital condition or acquired through injury such as from prolonged intubation. Surgeons face challenges in choosing the best SGS treatment for a particular patient because of variability in the success rate of each technique. Conventional open surgical resection and reconstruction have been proven effective but, in recent years, endoscopic surgery has become more prevalent as it eliminates the incision and reduces the surgery time and subsequent hospital stay. The purpose of this retrospective case study was to report on an endoscopic technique using a CO_2_ laser for cricotracheal stenosis resection (CTSR) for high-grade congenital SGS. From forty-five paediatric patients who underwent endoscopic intervention as a primary modality of treatment for high-grade SGS in a tertiary referral centre, a total of eight patients who met the inclusion criteria have been included in our study. This small patient series is the first to use a CO_2_ laser alone as a single excision tool to eliminate complex congenital SGS and restore airway patency. The procedure's goal was to return the airway to an early stage of postintubation injury prior to scar formation; therefore, surgical sessions at intervals of 2–3 weeks were performed to ensure natural epithelization, to remove any granulation tissue, and manage fibrosis. Successful treatment was defined as a resolution of symptoms, restoration of a normal patent airway with no stenosis, and decannulation. The success rate was 75%. Two outcomes need to be highlighted. First, the CO_2_ laser should be reconsidered as an excision tool for congenital SGS because of its low risk of exacerbating preexisting stenosis. It allows the surgeon to restore and augment the airway without the need for open surgery or dilatation. Second, the shorter interval between procedures is crucial for controlling the healing process and making sure that it is proceeding properly.

## 1. Introduction

The subglottic region is highly vulnerable to instrumentation and pathological processes. Children are either born with laryngotracheal stenosis or acquire it during their development. The subglottis plays a crucial role in children because it serves as the main site of airway narrowing [1]. Cricoid cartilage malformation is one of the major characteristics of stenosis in congenital cases. Prolonged intubation is a risk factor for noncongenital subglottic stenosis (SGS) and accounts for 90% of cases [2]. SGS treatment presents a significant challenge in children. Cricotracheal resection (CTR) or open reconstruction with laryngotracheal reconstruction (LTR) has been proven an effective and safe SGS treatment option for the paediatric population. However, the use of endoscopic techniques in the management of SGS treatment has grown over the last decades. Additionally, treatment of SGS in the paediatric population with less-invasive surgical procedures has advanced over time. Several endoscopic procedures such as stent placement, microdissection, endoscopic laser resection, and endoscopic dilation are applied either as a primary treatment strategy, or to compliment an open reconstructive procedure. These procedures offer three potential benefits: decrease in operation time, decrease in the admission length, and prevention of external incisions. The rate of success of the endoscopic approach is also increased by use of adjuvant treatments such as administration of mitomycin-C and steroids [3]. Surgeons face challenges in making decisions about the most efficient type of modality or the combination of modalities for SGS treatment due to variation in the success rates of each technique. Though no strict criteria for managing SGS have been codified, the endoscopic approach has been limited to low-grade (grades I and II), noncircumferential, and short-segment SGS [4–9]. In spite of all these recommendations and case selection, endoscopic management still shows recurrent scar formation rates of 40% to 70% [10]. In grade III SGS, the success rate declined to 13%, indicating that endoscopic treatment can seldom restore a subglottis to an anatomically normal state.

Studies have demonstrated only a minor role for laser treatment in pure congenital SGS except for a few trials of cricoid split and when using balloon dilatation [11]. The objective of this study was to describe an endoscopic technique using a CO2 laser for cricotracheal stenosis resection (CTSR) to manage high-grade complex SGS and to assess this technique in high-grade congenital SGS without open procedure. To the best of our knowledge, this is the first study to discuss the use of an endoscopic CO2 laser as a single excision tool to eliminate high-grade congenital SGS and restore airway integrity.

## 2. Materials and Methods

Ethical approval of the study was provided by the King Abdullah International Medical Research Centre (KAIMRC) with research protocol RC16/208/R.

### 2.1. Patients

In this retrospective case series, we reviewed the charts of 45 paediatric patients (ranging in age from one month to 14 years) who underwent endoscopic intervention as a primary modality of treatment for high-grade SGS in a tertiary referral centre (King Abdulaziz Medical City, Riyadh, Saudi Arabia) from January 1, 2011 to October 31, 2019. All children who had a workable diagnosis of congenital SGS and who underwent at least one endoscopic intervention as the primary treatment modality without previous primary open laryngotracheoplasty were included in the study. After applying our inclusion criteria, eight patients with congenital SGS out of 45 SGS cases managed by CO_2_ laser-assisted endoscopic CTSR at our institution were included in this study. The patients had been referred from different secondary hospitals in Saudi Arabia and were tracheostomized with a diagnosis of SGS based on direct laryngobronchoscopy (DLB) findings. Definitive or successful treatment was defined as a resolution of symptoms, restoration of a normal patent airway with no stenosis, and decannulation.

Children who were diagnosed with acquired SGS were excluded. Charts were reviewed for the number of primary surgeons, age, gender, history of prematurity, significant medical comorbidities, descriptions of SGS, types and timing of interventions for SGS, and airway status at the last available follow-up visit. The diagnosis and description of SGS were performed by the paediatric otolaryngologists in the operating room at the time of the direct laryngobronchoscopy (DLB). Information on the characteristics of the SGS was taken from the surgeon's description in the surgical notes. Three paediatric otolaryngologists, one consultant and two fellows, participated in the surgeries, and the consultant was always there to confirm the description. The Myer-Cotton grading system was used to standardize descriptions of the degree of the SGS. The follow-up lasted for at least three years after the last intervention.

### 2.2. Statistical Analysis

All collected data were analyzed with SPSS version 17 using the mean ± SD for the quantitative variables and frequencies and percent for the qualitative variables. All statistical analyses of the qualitative data before and after endoscopic treatment of SGS were performed using McNemar's test. Differences were considered to be significant when *p* was <0.05 and highly significant when *p* was <0.01.

### 2.3. Surgical Technique

**Figure 1:**
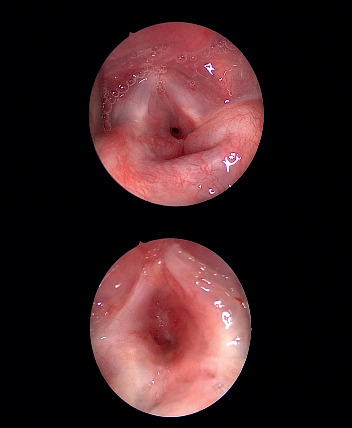
Congenital subglottic stenosis.

**Figure 2:**
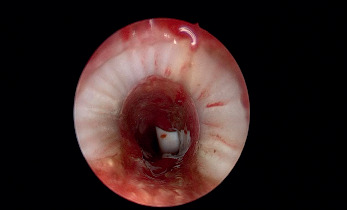
Excision of SGS by laser.

**Figure 3:**
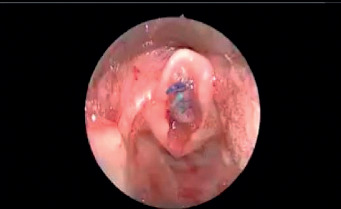
Insertion of endolaryngeal stent.

Under a spontaneous ventilation general anaesthesia technique, the patient's larynx was exposed using a rigid laryngoscope with lateral ports for ventilation. This technique permitted the surgical field to be free of ventilation material during the procedure. Endoscopy was undertaken to examine the area of stenosis and additional synchronous lesions. Photographic documentation of the stenotic segment was obtained using a 2.7 or 4 mm and 0-degree Karl Storz telescope. Descriptions of the grade, site, craniocaudal extension, distance from the vocal folds, and texture of the stenosis were crucial. The Cotton-Myer scale was used to estimate the grade of stenosis. A vocal cord spreader was then applied to expose the SGS (Figure 1). An UltraPulse® DUO CO2 surgical laser (Lumenis GmbH, Dreieichenhain, Germany) with a microspot, ultrapulse mode and a power of 125 mj/cm^2^ was used to excise the stenosis by peeling off the cricoid cartilage in a craniocaudal direction (Figure 2). The perichondrium of the cricoid cartilage is difficult to completely preserve but the aim was to avoid complete cartilage denudation (Figure 2). Mechanical dilatation is not part of this procedure. Haemostasis was achieved with pledgets soaked in adrenalin to keep the field bloodless. Submucosal injections of a long-acting corticosteroid, commonly triamcinolone acetonide (40 mg/ml), were done above and below the level of the excised stenosis. The laryngeal stent was then inserted and fixed by Lichtenberger needle to prevent restenosis by granulation tissue (Figure 3). The area was cooled and cleaned of any debris with saline-wetted pledgets. Patients were monitored in the paediatric intensive care unit for 24 to 48 hours using a noninvasive ventilation system. All patients received acetaminophen and nonsteroidal anti-inflammatory drugs to control pain. Systemic steroids (prednisolone, 1–2 mg/kg/day) were administered for ten days. Proton pump inhibitors (esomeprazole, 2 mg/kg/day) were prescribed for 28 days. Patients were fed postoperatively through a nasogastric tube and then gradually shifted to oral intake. The aim of this procedure was to return the airway to an early stage of postintubation injury prior to scar formation. Then, subsequent surgical sessions at intervals of 2–3 weeks were performed to remove the stent and to ensure that natural epithelization was proceeding favourably, to remove any granulation tissue and to manage early recurrent fibrosis. Mucosal healing from both edges was measured and documented regularly during all sessions until complete epithelization of the area occurred (Figure 2). Allowing scar tissue to accumulate and mature will negate the outcome and require the surgeon to start over from the beginning. After complete epithelization, the follow-up diagnostic laryngoscopy continued every six months for 48 months.

## 3. Results

After applying our inclusion criteria, only eight patients out of 45 SGS cases managed by CO_2_ laser-assisted endoscopic CTSR at our institution were included in this study. Four male and four female patients with an average age of 11.5 months (range, 0.1–24 months) were identified. Five patients had a history of concurrent pulmonary disease and three had a history of gastroesophageal reflux. Three patients reported neurological disease and one had congenital heart disease (Table 1). Presenting symptoms of SGS included severe dyspnoea, biphasic continuous stridor, dysphonia, and cough. Two patients (16%) were categorized as having idiopathic stenosis. When a quantitative airway diameter was reported, the Cotton-Myer grading scale was applied to estimate subglottic diameter. All eight patients (100%) had grade III stenosis (defined as 71%–99% stenosis). The craniocaudal extensions ranged from 8 mm to 15 mm with an average length of 11.6 mm (Table 2). All patients had circumferential stenosis and all of them had a tracheostomy surgery. The eight patients underwent 21 surgical procedures. Three of eight patients (37.5%) required a single procedure with a mean surgical duration of 1.4 hours. Five patients (62.5%) required multiple surgeries (3–4 procedures). Of the patients who required further procedures, the mean interval between surgeries was 3.1 weeks. The mean hospital stay was 4 days (range, 2–10 days) for each procedure.

Definitive or successful treatment was defined as a resolution of symptoms, restoration of a normal patent airway with no stenosis, and decannulation. Patients who have been treated successfully were decannulated within 4 to 5 weeks after the last surgical session with a mean of 31 days. The success rate was 75%. Two patients (25%) had residual grades I-II SGS and underwent open laryngotracheal reconstruction procedure with anterior and posterior grafts. Using the Friedman test to compare preoperative and stenotic grade showed statistical significance with a *p* value of 0.0082 (Table 2). All the patients who were decannulated were able to tolerate regular oral feeding. The median follow-up duration for all patients from the date of the first procedure to the last clinical visit was three years (range, 2–4.5 years).

## 4. Discussion

**Figure 4:**
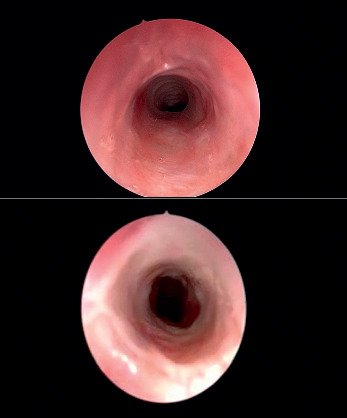
Endoscopic photographs showing the improvement in the extension of stenosis and the degree of severity in SGS that occurred in one patient during each surgical session of CTSR.

The subglottis, as a junction between two embryological growth centres, is particularly vulnerable to instrumentation and pathological processes [4]. It can be affected by a tenuous blood supply or microcirculation [12]. Other factors previously postulated to potentiate stenosis include exposure to gastric reflux, turbulent airflow, and presence of a complete cartilage ring [12]. SGS, regardless of its aetiology, is a difficult problem to treat. Treatment has traditionally involved open procedures such as LTR or PCTR [1, 2]. Although open procedures have high rates of decannulation [6], they also have the potential for severe complications [3, 13]. While endoscopic procedures avoid these complications, extreme variability in results is reported in the literature [10]. The recurrence of scar formation has shown high rates of 40% to 70% in endoscopic management [4]. To obtain good results, therefore, conservative endoscopic modalities have been advocated [4–8]. In 1982, Simpson et al. concluded that factors associated with poor endoscopic results or failure included circumferential scarring with cicatricial contracture and scarring wider than one cm in the vertical dimension [11]. Triglia et al. advised laser resection only for grade I SGS less than 5 mm in length [8]. Monnier et al. reviewed 100 patients treated solely by endoscopic means for laryngotracheal stenosis [14, 15]. The postoperative results showed that the improvement declined from 92% for grade I to 46% for grade II and 13% for grade III stenosis. When compared with open surgery for more severe grades of stenosis, the results of endoscopy were much less favourable [6]. It has been shown that CO_2_ lasers with pulsed technology vaporize tissue quickly, avoid thermal damage, permit constant tissue ablation, and avoid charring and scarring [14–16]. It was thought that laser energy would heat the surrounding tissues and might cause additional scar formation [9], but Hseu et al. reported in 2014 that CO_2_ laser surgery was not associated with worse outcomes or more frequent dilations [4]. This unprecedented finding encouraged the use of CO_2_ lasers not only for the radial incision but also as a “knife” to excise the tissue causing SGS. In addition, the suspension system of the CO_2_ laser (micromanipulator) is very sensitive, thereby allowing precise manipulation of the laser beam to resect the fibrosis *en bloc*. We achieved endoscopic excision of congenital SGS solely with a CO_2_ laser and ultimately augmented the airway without any mechanical dilatations. We did encounter one complication of minimal airway fire managed intraoperatively; also, the vocal cord shape in our series seemed more thickened and was covered by respiratory epithelium instead of stratified squamous epithelium. The healing process was monitored closely by performing therapeutic microlaryngoscopy every 2–3 weeks (Figure 4). Our findings concur with those of Hseu et al. that laser surgery was not associated with worse outcomes; on the contrary, there was obvious improvement [4]. We called this technique CTSR. In CTSR, the fibrous stenotic portion was resected as in open PCTR, but without affecting the integrity of the anatomical framework, and the lumen of the airway was augmented and restored as in open LTR with a minimally invasive endoscopic technique.

The present study is a preliminary report to assess the technique for correcting high-grade circumferential SGS. Despite the selection criteria, we achieved a success rate of 75% and only two patients (25%) continued to present residual grade II SGS. Reports in the literature following primary endoscopic treatment of LTS showed success rates from 40% to 94%, depending on the appropriateness of the indication [4–13]. The number of subjects in this report was limited; therefore, some comparisons will be underpowered. The average number of surgical sessions required for each patient was three. 38% of patients required only a single procedure and 62% required multiple surgeries (3–4 procedures), which are comparable to the results of other studies that were more conservative in using CO_2_ lasers [4]. One of the drawbacks of our approach is the need for multiple surgical sessions with its consequences of psychological trauma to paediatric patients although this can be balanced with the shorter hospital stay and less-invasive care compared to other open techniques. The major difference in this technique is the interval between surgical sessions. The anticipated interval between procedures is set at 2–3 weeks to control the natural healing process, remove granulation tissue, and manage recurrent stenosis. In other algorithms, it sometimes takes months before the patient presents in need of urgent intervention [4]. In our opinion, this interval is too long. It can negate the beneficial outcomes of the surgery and cause the trachea to revert to its previous degree of stenosis or worse. In spite of the comorbidities in some patients, which might be contraindications to open surgery, this technique was performed successfully. Of course, the number of subjects was small, owing to the rarity of the disease and the selection criteria, but it is considered as a preliminary report to assess this technique in acquired high-grade, circumferential SGS endoscopically. This small patient series is the first to use a CO_2_ laser alone as a single excision tool to eliminate complex SGS and restore airway patency. Finally, two outcomes need to be highlighted. First, the CO_2_ laser should be reconsidered as an excision tool for SGS without much risk of worsening preexisting stenosis. It provides a chance to restore and augment the airway without the need for open surgery or dilatation. Second, the shorter interval between procedures is crucial for controlling the healing process and making sure that it is proceeding properly.

## 5. Conclusions

SGS is a difficult problem for the otolaryngologist to address and is complicated by the fact that there is no standard algorithm for treatment. This study was designed to assess a new endoscopic technique using a CO_2_ laser to resect cricotracheal stenosis without affecting the airway framework integrity. In this study, it was used to treat high-grade, circumferential SGS. The success rate of this small sample is promising. This is the first patient series reported to date using a CO_2_ laser as the sole excision tool to eliminate SGS without any mechanical dilatation. This innovative surgical approach might change the consensus for the endoscopic surgical management of SGS. Further larger studies are needed to confirm its efficacy and safety.

## Figures and Tables

**Table 1:** Demographics, length of stenosis, preop and postop grade of SGS, medical Hx, and number of procedures (patients undergoing open surgery marked in red).

	Age at diagnosis	Current age	Gender	Length of stenosis (mm)	SGS preop	SGS postop	Medical HX	Number of procedures
Pt#1	11 months	5.4 years	F	9	3	0	Stickler syndrome	1
Pt#2	12 months	4.2 years	M	8	3	2	Neurological hypotonia	4
Pt#3	15 months	3.3 years	F	12	3	1	None	1
Pt#4	5 months	2.8 years	M	13	3	1	Cervical teratoma	3
Pt#5	24 months	4 years	M	10	3	1	Neurological disease	1
Pt#6	16 months	5 years	F	12	3	0	Neurological disease	3
Pt#7	9 months	4 years	M	15	3	2	Cardiac disease	4
Pt#8	1 month	4 years	F	14	3	0	None	4

**Table 2:** Stridor and endoscopic demographic grading of SGS before and after CTSR.

Variable	Before CTSR	After CTSR	*p* value
Stridor			
No	0 (0.0%)	6 (75%)	
Yes	8 (100.0%)	2 (25%)	<0.01

Grade of stenosis			
Grade 0	0	6 (75%)	
Grade I	0	0	
Grade II	0	2 (25%)	
Grade III	8 (100%)	0	<0.01
Grade IV	0	0	

## Data Availability

The data used to support the findings of this study are available from the corresponding author upon request.
